# The Chronic Metabolic Fingerprint of Canine Anxiety: Hair Trace Element Dyshomeostasis and the Dissociation of Behavioral Recovery via *Hericium erinaceus*

**DOI:** 10.1007/s12011-026-05107-4

**Published:** 2026-05-04

**Authors:** Çağın Çevik, Bahar Ozturk Kurt, Bengü Bilgiç, Mehmet Erman Or, Alev Meltem Ercan

**Affiliations:** 1 Department of Biophysics, Cerrahpaşa Faculty of Medicine, İstanbul University-Cerrahpaşa, İstanbul, 34098 Turkey; 2 Department of Internal Medicine, Faculty of Veterinary Medicine, İstanbul University- Cerrahpaşa, İstanbul, 34320 Turkey

**Keywords:** Canine Anxiety, Hair Trace Elements, Chronic Stress Biomarkers, *Hericium erinaceus*, Nutraceutical Therapy, Metabolic Stress

## Abstract

Canine anxiety is a complex welfare issue involving neuro-endocrine dysregulation. While acute stress biomarkers are widely used, the chronic metabolic consequences of anxiety remain under-investigated. Hair mineral analysis offers a retrospective window into long-term systemic homeostasis. This study aimed to identify chronic trace element signatures in the hair of anxious dogs compared to healthy controls and evaluate whether behavioral recovery induced by *Hericium erinaceus* supplementation is reflected in the hair mineral matrix. Hair samples from 21 healthy controls and 8 clinically anxious dogs were analyzed for a 16-element panel (Zn, Cu, Fe, Mg, Mn, Se, Ni, Si, Ca, P, Al, Cd, Cr, As, B, and Pb) using ICP-OES. The anxious cohort received *H. erinaceus* extract (1000 mg/10 kg) daily for 28 days. Anxious dogs exhibited a distinct dyshomeostatic profile characterized by significantly higher hair zinc and aluminum, alongside a profound depletion of nickel (*p* < 0.001) and significantly elevated phosphorus (P) levels (*p* = 0.003). Supplementation led to a substantial 62% reduction in anxiety scores (*p* = 0.002), and post-treatment scores were no longer statistically different from those of the control group. However, most hair mineral concentrations remained stable post-treatment, with the notable exception of a significant increase in calcium (Ca) levels (*p* = 0.012). Furthermore, Spearman’s correlation analysis revealed that higher anxiety severity was strongly and negatively associated with hair iron (Fe) and chromium (Cr) levels (*r*_*s*_ = -0.77, *p* = 0.041). These findings suggest that hair Zn, Al, and Ni status may represent potential candidate biomarkers, warranting validation in larger cohorts of the chronic anxious phenotype. The persistence of these signatures despite rapid clinical improvement supports a temporal dissociation between behavioral recovery and metabolic normalization as reflected in the hair matrix.

**Graphical Abstract:**

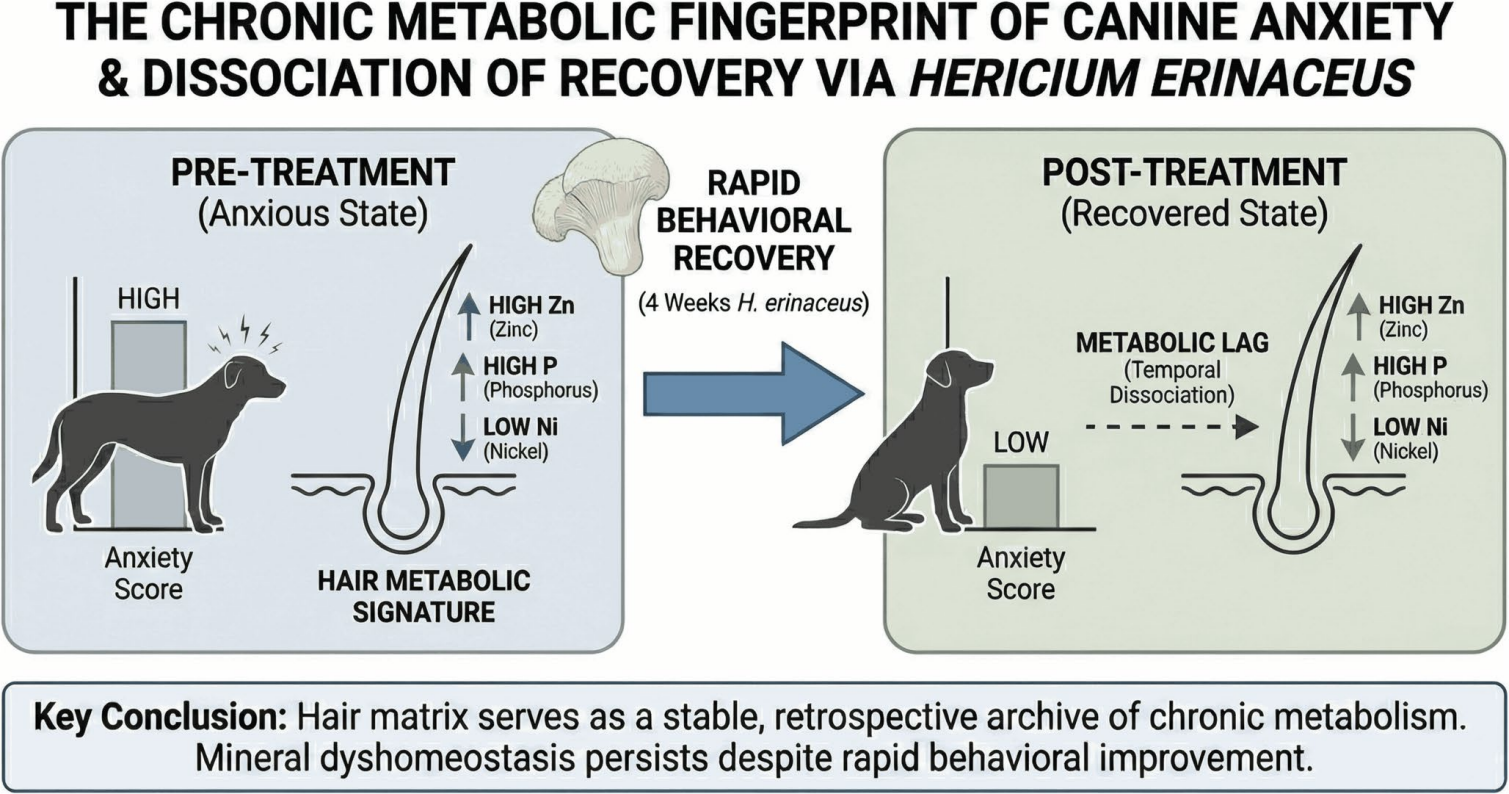

## Introduction

 Anxiety in domestic dogs represents a multifaceted welfare challenge characterized not only by maladaptive behavioral responses, such as persistent fear, noise sensitivity, and separation distress, but also by profound underlying physiological dysregulation. While the behavioral manifestations of canine anxiety are well-documented, the chronic metabolic consequences of prolonged stress remain less understood. It is well established that psychological stress triggers the hypothalamic-pituitary-adrenal (HPA) axis, leading to oxidative stress and neuroinflammation [1, 2]. Traditionally, acute stress assessment in veterinary medicine has relied on fluid matrices, such as serum or salivary cortisol. However, these markers are susceptible to circadian rhythms, pulsatile secretion, and immediate handling stress, often providing only a transient “snapshot” of the animal’s physiological state rather than a longitudinal record of welfare [3, 4].

To overcome the limitations of acute fluid sampling, hair analysis has emerged as a robust non-invasive tool for biomonitoring chronic metabolic status. Unlike blood, which is tightly regulated by homeostatic mechanisms to maintain stable mineral concentrations even during systemic deficits, the hair shaft incorporates trace elements into its keratin matrix during growth [5]. This process effectively creates a retrospective “calendar” of systemic mineral availability and neuro-endocrine activity over weeks or months. Consequently, hair mineral profiles offer a stable archive of the organism’s response to chronic stressors, unaffected by the immediate excitement of clinical sampling [6]. Despite these advantages, hair analysis presents certain limitations that must be acknowledged. External contamination from environmental exposure or grooming products may influence elemental content despite standardized washing procedures. Additionally, inter-individual variability in hair growth rates, breed-specific coat characteristics, and pigmentation may affect mineral incorporation dynamics. Therefore, hair mineral analysis should be interpreted as a retrospective biomarker of chronic exposure rather than an absolute indicator of real-time systemic mineral status [7, 8].

Trace elements and minerals act as critical cofactors for enzymatic systems governing neurotransmitter synthesis, including GABA and serotonin, and antioxidant defense mechanisms [9]. Chronic activation of the HPA axis is hypothesized to induce systemic mineral redistribution or “dyshomeostasis” to meet elevated metabolic demands. For instance, zinc (Zn) is essential for the modulation of glutamatergic and GABAergic synapses, and its systemic fluctuation has been linked to anxiety-like behaviors in mammalian models [10]. While some elements like Zn, Fe, and copper (Cu) are vital cofactors, others like nickel (Ni) and aluminum (Al) are of interest due to their potential neurotoxic effects or interference with essential mineral metabolism during chronic psychological strain. Recent studies in canine neurology have suggested that neurological instability leaves a discernible “elemental signature” in the hair matrix [11]. However, the specific chronic trace element profile associated with the anxious canine phenotype remains under-investigated.

Parallel to diagnostic advancements, natural therapeutic interventions targeting the gut-brain and neurotrophic axes are gaining prominence. *H. erinaceus* (Lion’s Mane), a medicinal mushroom, has demonstrated significant neuroprotective and anxiolytic properties [12]. These effects are primarily attributed to bioactive compounds such as hericenones and erinacines, which can cross the blood-brain barrier and stimulate Nerve Growth Factor (NGF) synthesis [13, 14]. While its behavioral efficacy is documented, it is currently unknown whether the clinical recovery induced by *H. erinaceus* is accompanied by an immediate modulation of the chronic metabolic footprint recorded in hair, or if a temporal dissociation exists between behavioral improvement and hair mineral normalization.

This study aims to bridge this gap by addressing two primary objectives: (1) to identify whether anxious dogs exhibit a distinct chronic hair trace element profile (a “metabolic fingerprint”) compared to healthy controls, by characterizing a broad 16-element panel (Zn, Cu, Fe, Mg, Mn, Se, Ni, Si, Ca, P, Al, Cd, Cr, As, B, and Pb); and (2) to evaluate whether behavioral recovery following a 4-week *H. erinaceus* supplementation serves to acutely normalize these mineral markers. We hypothesized that anxious dogs exhibit alterations in hair mineral profiles reflecting chronic stress, and that behavioral recovery may not immediately correspond to changes in the hair matrix due to the physiological dynamics of hair growth and mineral incorporation.

## Materials and Methods

### Ethical Approval and Study Population

This study was conducted in strict accordance with animal welfare guidelines and approved by the Ethics Committee of Istanbul University-Cerrahpasa Faculty of Veterinary Medicine (Approval No: 2021/36). The study population comprised 29 client-owned mixed-breed dogs, stratified into a healthy Control Group (n = 21) exhibiting no history of behavioral pathologies and an Anxious Group (n = 8) diagnosed with clinically relevant anxiety disorders. Inclusion criteria for the anxious group were established through standardized behavioral assessments involving owner-completed questionnaires validated for canine anxiety. Dogs were excluded if they had chronic systemic diseases, current infections, recent mineral supplementation within 3 months, or chronic medication use that could affect mineral metabolism. Demographic and clinical characteristics of the study population are summarized in Table 1.

**Table 1:** Demographic and clinical characteristics of the study population

Parameter	Control Group (*n* = 21)	Anxious Group (*n* = 8)	*p*-value
**Age (years**,** Mean ± SD)**	4.5 ± 2.1	5.1 ± 1.9	0.56
**Body Weight (kg**,** Mean ± SD)**	18.9 ± 7.4	16.8 ± 6.5	0.35
**Sex (Male/Female)**	10/11	4/4	1.00
**Breed Distribution** **Geographical Area** **Dietary Intake**	Mixed Breed (100%) Metropolitan Area Standard Commercial Diet	Mixed Breed (100%) Metropolitan Area Standard Commercial Diet	- - -

Footnote: Data are presented as mean ± standard deviation (SD). P-values for continuous variables were calculated using the Mann–Whitney U test, and for categorical variables using Fisher’s exact test. All dogs were mixed-breed, lived in similar metropolitan environments, and were fed standard commercial diets, minimizing potential confounding effects of environmental and dietary variability on trace element profiles

### Experimental Design and Supplementation Protocol

A prospective, open-label interventional design was employed for the anxious cohort. Dogs in the Anxious Group received a standardized oral supplementation of *H. erinaceus* extract at a dosage of 1000 mg per 10 kg of body weight daily for a period of 28 consecutive days. Clinical assessments and biological sampling were performed at two distinct time points, immediately prior to the initiation of treatment (PRE) and upon completion of the 28-day supplementation period (POST). The Control Group was sampled once to establish baseline reference intervals for hair trace element concentrations.

### Hair Sample Collection and Preparation

To ensure consistency and minimize environmental contamination, hair samples of approximately 0.5 g were shaved from the dorsal nuchal region using stainless steel clippers. Samples were strictly stored in polyethylene bags to prevent external mineral interaction. Prior to analysis, all samples underwent a rigorous sequential washing protocol to remove exogenous contaminants such as sebum, dirt, and environmental particulates. Hair samples were washed according to the procedure described by Draghi et al. [6]. Briefly, samples were washed in acetone, followed by three rinses with deionized water and a final rinse with acetone to remove exogenous contaminants.

### Sample Mineralization and Elemental Analysis (ICP-OES)

The prepared hair samples (0.2 g) were subjected to wet acid digestion to solubilize the keratin matrix. Mineralization was performed in a controlled laboratory oven at 180 °C using a high-purity mixture of nitric acid (HNO₃, 65%) and perchloric acid (HClO₄, 70%) in a 2:1 ratio. This specific acid combination was selected to ensure the complete oxidation of organic matter and the liberation of bound minerals from the hair keratin. Following the mineralization process, the clear digests were diluted to a final volume with deionized water.

Elemental analysis was performed using Inductively Coupled Plasma-Optical Emission Spectrometry (ICP-OES; Thermo iCAP 6000 series, Thermo Fisher Scientific) at the Istanbul University-Cerrahpasa. A panel of 16 essential and non-essential elements was quantified, including Cu, Fe, Mg, Mn, Se, Zn, Ni, Si, Ca, P, Al, Cd, Cr, As, B, and Pb. Concentrations were calculated and expressed as parts per million (ppm, µg/g dry weight).

### Quality Control and Analytical Performance

Analytical accuracy and precision were maintained using high-purity standard reference solutions for each element to generate multi-point calibration curves. Because certified hair reference material was not available for this analytical setup, method performance was verified through recovery testing and procedural blanks. To verify the efficiency of the digestion and measurement process, recovery tests were performed using spiked samples. The recovery rates ranged between 92% and 106%, confirming the robustness of the methodology.

### Statistical Analysis

Data distribution was assessed using the Shapiro-Wilk test. Due to the skewed distribution and high variability observed in hair trace element concentrations, results are expressed as Median and Interquartile Range (IQR). Differences across the three groups (Control, PRE, and POST) were analyzed using the non-parametric Kruskal-Wallis test, followed by Dunn’s post-hoc test for pairwise comparisons with Bonferroni correction. The relationship between hair mineral profiles and total anxiety scores, combining the Control and PRE groups, was evaluated using Spearman’s rank-order correlation. Statistical significance was defined as *p* < 0.05. Group comparisons for demographic variables were performed using the Mann–Whitney U test for continuous variables and Fisher’s exact test for categorical variables, due to the small sample size and non-normal distribution of the data.

Data analysis was conducted using the Python programming language (v3.9), utilizing the Scipy and Pandas libraries for statistical computing. Behavioral efficacy was evaluated using the Wilcoxon signed-rank test to compare total anxiety scores within the anxious cohort (PRE vs. POST). Although the anxious cohort size was limited (*n* = 8), the primary biomarkers exhibited large and consistent effect magnitudes with minimal intra-group variance, which reduces the likelihood of Type II error and supports the robustness of the observed mineral dyshomeostasis despite the exploratory nature of the study.

## Results

No significant differences were observed between the Control and Anxious groups in terms of age, body weight, or sex distribution (*p* > 0.05 for all comparisons), indicating a demographically comparable study population (Table 1).

### Behavioral Validation of Anxiety and Clinical Efficacy

**Fig. 1:**
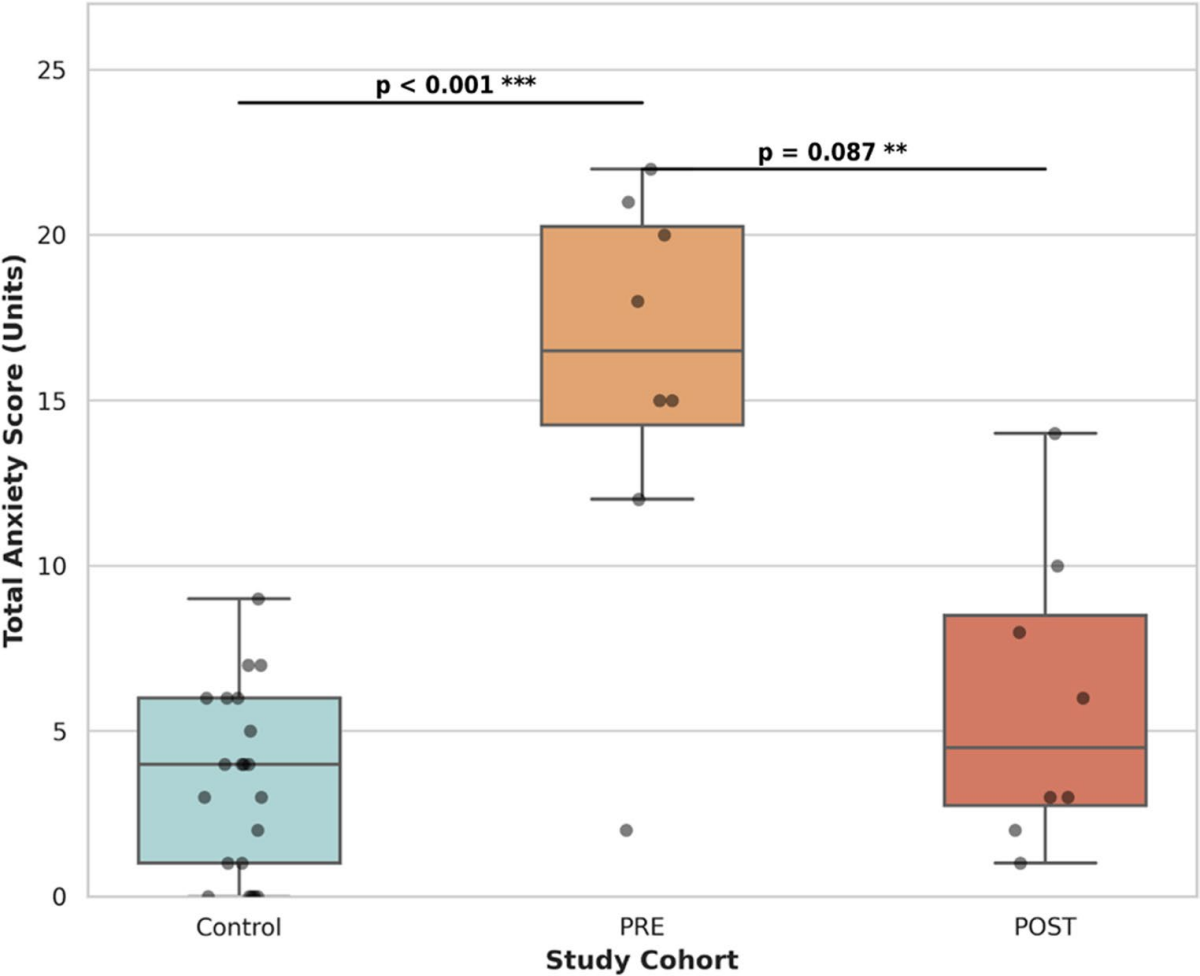
Comparison of Total Anxiety Scores Across Experimental Groups. The figure displays the distribution of Total Anxiety Scores for the healthy Control group (*n* = 21), the anxious cohort at baseline (PRE, *n* = 8), and following 4 weeks of H. erinaceus supplementation (POST, *n* = 8). Boxplots represent the Median and Interquartile Range (IQR), with individual data points overlaid as a strip plot. Statistical analysis revealed a highly significant difference between the Control and PRE-treatment groups (*p* < 0.001), confirming the clinical anxiety status. Following treatment, a significant reduction in scores was observed (*p* = 0.002), while POST scores were not significantly different from those of the healthy control group (*p* = 0.087)

Baseline behavioral assessments confirmed the clinical status of the study population. The Anxious Group (PRE) exhibited significantly higher total anxiety scores, with a Median of 16.0 (IQR: 9.2), compared to the healthy Control Group, which had a Median of 3.0 (IQR: 4.0) (*p* < 0.001) (Fig. 1). Following the 28-day supplementation with *H. erinaceus*, a substantial behavioral improvement was recorded within the anxious cohort. Total anxiety scores decreased by approximately 62% to a Median of 6.0 (IQR: 6.5).

The Wilcoxon signed-rank test confirmed that this reduction was statistically significant (*p* = 0.002). Notably, the post-treatment scores (POST) did not differ statistically from those of the healthy Control group (*p* = 0.087), indicating that the behavioral profile improved substantially after the 4-week intervention and was no longer statistically different from that of the non-anxious population (Fig. 1).

### Chronic Hair Mineral Signatures of the Anxious Phenotype

Comparative analysis of the hair mineral matrix revealed a distinct chronic metabolic footprint associated with canine anxiety. Statistical dysregulation was observed in 7 of the 16 analyzed elements (Table 2). Anxious dogs (PRE) demonstrated a profound dyshomeostasis characterized by significantly higher hair Zn (Median: 213 ppm) and Al (Median: 49.8 ppm) compared to healthy controls (*p* < 0.001). Conversely, hair Ni levels were severely depleted in the anxious cohort, with a Median of 0.08 ppm relative to 0.81 ppm in controls (*p* < 0.001).

**Fig. 2:**
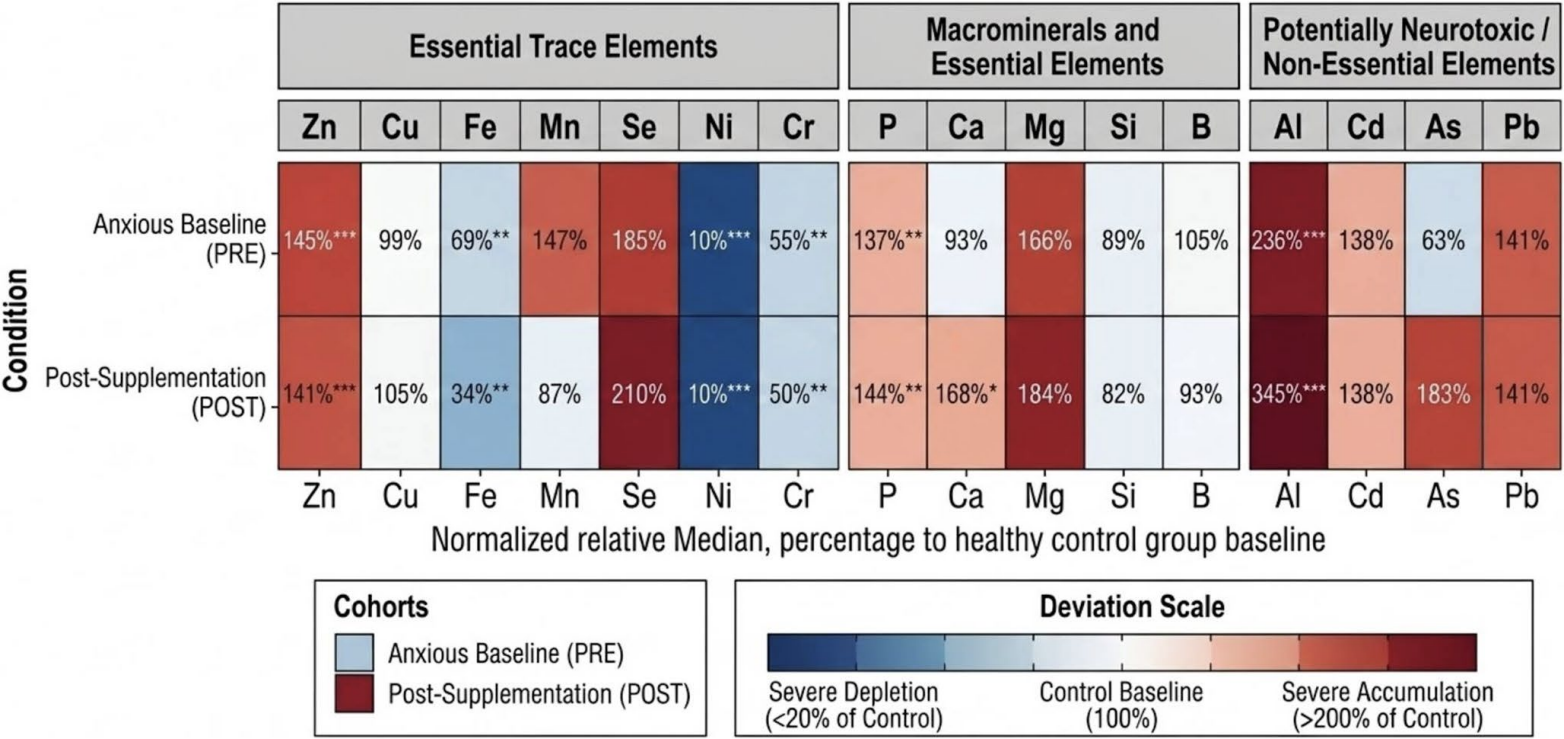
Metabolic Heatmap of Relative Hair Trace Element and Mineral Deviations in Anxious Dogs. The heatmap visualizes the normalized relative Median concentrations of the complete 16-element panel in the anxious-baseline (PRE) and *H. erinaceus*-supplemented (POST) cohorts relative to the healthy control group (baseline = 100%). Elements are categorized into essential trace elements, macrominerals/essential elements, and potentially neurotoxic/non-essential elements. Color intensity represents the magnitude of metabolic deviation, with red indicating increased accumulation and blue indicating depletion. Detailed Median, IQR, pairwise group comparisons, and overall p-values for all elements are provided in Table 2. The heatmap is intended as a visual summary of relative directional changes across the mineral panel

Significant elevations were also noted for P (*p* = 0.003) and Ca (*p* = 0.012) in the anxious population (Table 2). While broad elemental dysregulation was evident, the magnitude of deviation for Zn and Ni relative to controls exceeded 40% and 70%, respectively, suggesting that these elements may represent candidate chronic biomarkers of anxiety-related metabolic stress. The relative directional deviation of the complete 16-element panel from the healthy baseline is visually summarized in the metabolic heatmap (Fig. 2).

### Correlation between Behavioral Severity and Mineral Profiles

Spearman’s rank-order correlation analysis was conducted to evaluate the relationship between the intensity of clinical symptoms and the chronic mineral record. In the combined cohort comprising the Control and PRE groups, total anxiety scores exhibited a strong and significant negative correlation with hair Fe (*r*_*s*_ = -0.77, *p* = 0.041) and Cr (*r*_*s*_ = -0.77, *p* = 0.041). These findings indicate that higher anxiety severity is objectively mirrored by a greater depletion in these specific hair trace elements. No other significant correlations were found within the 16-element panel (*p* > 0.05).

### Stability of the Hair Matrix Footprint Post-Treatment

Despite the rapid and significant behavioral recovery observed after 28 days of *H. erinaceus* supplementation, the hair mineral profile remained largely stable. No statistically significant differences were found in the concentrations of Zn, Ni, P, Al, Fe, or Cr between the PRE and POST treatment stages (*p* > 0.05) (Table 2). For instance, hair Zn levels remained elevated (Median: 208 ppm) and Ni levels remained suppressed (Median: 0.08 ppm) in the POST group.

One notable exception was hair Ca, which showed a significant increase in the POST group (Median: 1782 ppm) compared to the PRE stage (Median: 982 ppm) (*p* = 0.012). This lack of acute fluctuation for the primary biomarkers confirms that while the intervention induced rapid neuro-behavioral recovery, the hair matrix largely retained the metabolic footprint of the preceding chronic anxious state during the immediate therapeutic window.

**Table 2:** Hair trace element and mineral concentrations (ppm) in healthy and anxious dogs

Element (ppm)	Control (*n* = 21)	PRE (*n* = 8)	POST (*n* = 8)	*P*-value (Overall)
Zn	147 (35)^a^	213 (11)^b^	208 (44)^b^	< 0.001
Ni	0.81 (0.40)^a^	0.08 (0.40)^b^	0.08 (0.15)^b^	< 0.001
P	230 (103)^a^	316 (35)^b^	332 (53)^b^	0.003
Al Fe Ca Cr Cu Mg Mn Se Si B Pb Cd As	21.1 (19.4)^a^ 267 (287)^a^ 1060 (419)^a^ 4.06 (1.34)^a^ 20.3 (8.1) 77.1 (59.9) 1.79 (2.43) 1.00 (1.18) 110 (52) 359 (167) 0.17 (0.24) 0.08 (0.06) 0.54 (0.88)	49.8 (27.1)^b^ 185 (116)^ab^ 982 (129)^a^ 2.22 (2.02)^b^ 20.1 (3.2) 128 (65) 2.63 (2.61) 1.85 (1.01) 97.8 (61.7) 378 (180) 0.24 (0.23) 0.11 (0.09) 0.34 (2.15)	72.9 (36.3)^b^ 89.5 (60.8)^b^ 1782 (460)^b^ 2.04 (1.32)^b^ 21.4 (3.8) 142 (53) 1.56 (1.88) 2.10 (1.76) 90.2 (26.2) 334 (276) 0.24 (0.23) 0.11 (0.09) 0.99 (1.17)	< 0.001 0.005 0.012 0.001 0.992 0.199 0.353 0.118 0.943 0.709 0.629 0.075 0.772

Footnote: Data are presented as Median (Interquartile Range). p-values represent the significance of differences across the three groups calculated via the Kruskal-Wallis test. Superscript letters (a, b) indicate significant pairwise differences (*p* < 0.05) evaluated via Dunn’s post-hoc test with Bonferroni correction. Statistical significance was strictly defined as *p* < 0.05

## Discussion

The present study elucidates a distinct chronic mineral “signature” associated with the anxious canine phenotype and demonstrates a temporal dissociation between behavioral recovery and metabolic normalization. Our findings reveal that while *H. erinaceus* supplementation effectively mitigates anxiety-related behaviors within four weeks, it does not acutely alter the long-term mineral record sequestered in the hair matrix. These results provide critical insights into the neuro-metabolic environment of canine anxiety, suggesting that behavioral symptoms and systemic mineral dyshomeostasis operate on different physiological time scales. The trace element analysis in the present study was conducted following the validated hair biomonitoring protocol for domestic animals established by Draghi et al. [6], utilizing the same sample mineralization and ICP-OES measurement parameters.

The identification of significantly elevated hair Zn concentrations in anxious dogs (213 ppm vs. 147 ppm in controls) presents a compelling contrast to the hypozincemia typically reported in acute stress models. This “Zinc Paradox,” characterized by high tissue/hair accumulation despite potential serum depletion, likely reflects a chronic compensatory mechanism involving Metallothioneins (MTs). MTs are stress-inducible proteins that bind physiological Zn to protect cells against oxidative damage [15]. Under conditions of chronic HPA-axis activation, the upregulation of MT expression leads to an increased sequestration of Zn from the circulation into keratinized tissues [16]. Thus, the elevated hair Zn observed in this study may reflect not dietary excess, but rather a retrospective signal of prolonged oxidative stress adaptation. This concept of stress-driven mineral redistribution is further supported by human studies where altered Zn homeostasis and Zn-Cu ratios have been consistently linked to generalized anxiety and mood disorders [9, 17].

A novel and significant finding of this study is the profound depletion of hair Ni in the anxious cohort (0.08 ppm) compared to healthy controls (0.81 ppm). Nickel is not widely recognized as an essential trace element in mammalian enzymatic systems; however, excessive or deficient exposure has been associated with the modulation of oxidative stress pathways and interference with enzymatic activity [18]. Given the established bidirectional communication between the gut microbiome and the brain in anxiety disorders [19], the depletion of Ni may indicate a systemic exhaustion of trace elements required for microbial homeostasis during chronic psychological strain. This finding is compatible with the hypothesis that canine anxiety may involve disruption of the microbiota-gut-brain axis; however, this interpretation remains speculative and requires direct mechanistic investigation. Our analysis also revealed a significant elevation of P in the hair of anxious dogs (316 ppm) relative to controls (230 ppm). P accumulation likely reflects the catabolic impact of chronic glucocorticoid exposure. Prolonged elevation of cortisol is known to impair osteoblast function and stimulate osteoclast-mediated bone resorption, releasing Ca and phosphate from skeletal stores into the systemic circulation [20]. Consequently, the increased deposition of P in the hair shaft may represent the “metabolic debris” of stress-induced bone turnover, highlighting the systemic physiological toll of untreated anxiety.

Perhaps the most concerning finding is the significant accumulation of Al in the anxious group (49.8 ppm vs. 21.1 ppm). Unlike Zn or P, Al is a non-essential, neurotoxic element capable of inducing oxidative stress, neuroinflammation, and blood-brain barrier dysfunction [21, 22]. The presence of elevated Al in the hair of anxious dogs may reflect altered mineral handling during chronic stress and/or differences in systemic exposure or deposition dynamics. Furthermore, this accumulation aligns with models of metal-induced neurotoxicity, where Al promotes lipid peroxidation in the hippocampus, a brain region central to anxiety regulation. Interestingly, hair Al concentrations remained high in the POST group despite clear behavioral improvement. One plausible explanation is that hair reflects earlier metabolic conditions, as trace elements become incorporated during growth and remain relatively stable once keratinized. In this context, the observed elevation may represent prior systemic stress rather than current physiological status. It is also possible that recovery-related metabolic adjustments or redistribution processes influence how Al is deposited in hair. Longer-term follow-up beyond the four-week intervention would help clarify whether mineral levels eventually parallel behavioral remission.

Selenium (Se) exhibited a relative increase in the POST group, which may suggest a delayed adaptive antioxidant response given its role in glutathione peroxidase activity and neuroprotection. Although P levels were significantly elevated in the PRE and POST anxious groups compared with controls, the absolute concentrations remained within physiologically acceptable ranges for canine hair mineral composition. Therefore, the observed increase may reflect a relative shift in chronic mineral handling rather than an overt pathological excess. Given the tightly regulated physiological interplay between Ca and P, interpretation of these changes should remain cautious. In our dataset, Ca also differed significantly across groups, with the highest values observed in the POST stage; however, this pattern did not track acute behavioral improvement and is more consistent with delayed mineral incorporation into the hair shaft than with immediate systemic normalization. The 62% reduction in total anxiety scores following *H. erinaceus* supplementation supports the supplement’s anxiolytic potential. Mechanistically, this recovery is attributed to the neurotrophic activity of hericenones and erinacines, which stimulate Nerve Growth Factor (NGF) synthesis and promote hippocampal neurogenesis [12, 23].

Crucially, the behavioral normalization observed in this study occurred without a concurrent shift in hair mineral profiles. This stability supports the utility of hair as a metabolic archive rather than a real-time monitor. Canine hair grows at an approximate rate of 0.6 to 1 cm per month, a rate that varies significantly by breed and season [24]. Consequently, the hair shaft collected after the 28-day treatment period predominantly reflects the mineral incorporation of the preceding months rather than the immediate therapeutic window. This finding supports the view that hair mineral analysis is relatively stable against short-term fluctuations, serving as a retrospective indicator of the animal’s chronic history rather than its acute state.

Importantly, hair mineral analysis should not be interpreted as a tool for acute clinical decision-making. Instead, it reflects a longitudinal integration of systemic mineral availability over the hair growth cycle, reinforcing its role as a retrospective biomarker of chronic physiological history. To contextualize the findings, the mineral concentrations observed in the healthy control group were considered alongside published values reported for non-pathological dogs. In particular, the Median concentrations for Zn (147 ppm) and P (230 ppm) in the healthy cohort were broadly consistent with previously reported physiological ranges, supporting the use of the control group as a reasonable comparative baseline.

Growing evidence from both veterinary and human medicine supports the association between trace element metabolism and anxiety-related phenotypes. In human studies, alterations in Zn homeostasis have been consistently linked to anxiety and mood disorders. Reduced circulating Zn levels and altered Zn-Cu ratios have been reported in individuals with generalized anxiety and depressive symptomatology [9]. Zinc plays a critical modulatory role in glutamatergic and GABAergic neurotransmission, and experimental Zn deficiency has been shown to induce anxiety-like behaviors in animal models. These findings support the concept that chronic stress may disrupt Zn redistribution pathways, leading to measurable peripheral mineral alterations [9, 17, 25].

Experimental studies in mice have demonstrated that chronic restraint stress increases metallothionein expression and modulates Zn transporter activity in association with oxidative stress pathways. Such alterations indicate that stress may actively reshape Zn handling at the tissue level, supporting the concept of stress-driven mineral redistribution [26]. Altered handling of non-essential metals has also been implicated in neuropsychiatric conditions in humans, including anxiety-related disorders [27, 28].

The significant negative correlation identified between hair Fe and Cr levels and total anxiety scores (r_s_ = -0.77, *p* = 0.041) suggests that these elements may be associated with chronic stress intensity. Fe is an essential cofactor for tyrosine hydroxylase, influencing dopamine and norepinephrine synthesis. Its depletion, alongside elevated Al, which is a known neurotoxic pro-oxidant, highlights the profound metabolic shift associated with the anxious phenotype [8].

While these findings are promising, the study is limited by the small sample size of the anxious cohort (*n* = 8), which warrants caution in generalizing the results to the broader canine population. Future longitudinal studies extending beyond 3 to 6 months are necessary to determine the time course required for the hair mineral fingerprint to normalize following behavioral recovery. Additionally, although all dogs were fed standard commercial diets, strict standardization of dietary intake was not feasible in this client-owned population. However, the use of a control group from the same geographical region minimizes the impact of environmental trace element variations. Potential confounding factors such as environmental exposures, passive tobacco smoke, and subtle dietary differences could not be fully controlled. Furthermore, the absence of longitudinal hair segmentation analysis limits the temporal resolution of mineral incorporation. In addition, serum or plasma trace element measurements were not available in the present study; therefore, acute circulating mineral status could not be directly compared with the retrospective mineral archive represented by hair. These findings should be interpreted within the limitations of the small sample size and require validation in larger and longitudinal cohorts.

Finally, it is crucial to note that the distinct mineral dyshomeostasis, specifically high Zn and low Ni, was already established at baseline (PRE) prior to any supplementation, confirming the profile reflects the endogenous metabolic state of the anxious phenotype rather than an exogenous dietary confounding factor. Furthermore, the lack of significant fluctuation in post-treatment mineral levels supports the premise that the extract did not introduce a supranutritional mineral load capable of skewing the hair analysis results. Longitudinal studies extending beyond multiple hair growth cycles are required to determine the precise timeframe needed for normalization of the chronic mineral fingerprint following behavioral recovery.

## Conclusion

This study suggests that canine anxiety is associated with distinct mineral alterations that are preserved within the hair matrix as a chronic metabolic signature. In anxious dogs, this profile was characterized by elevated Zn, P, and Al levels, together with marked depletion of Ni. Comparison of the healthy cohort with published veterinary data supported the use of the control group as a reasonable baseline for interpretation. Overall, these findings are consistent with the view that chronic stress may influence long-term mineral handling and deposition in hair. The observed Ni depletion may also reflect broader metabolic or microbiota-related alterations, although this interpretation requires further mechanistic study.

Our longitudinal findings further support a temporal dissociation between clinical and metabolic recovery. Although *H. erinaceus* supplementation was associated with a 62% reduction in behavioral anxiety scores within four weeks, this improvement was not accompanied by acute normalization of the hair trace element profile. These findings support the interpretation that hair functions primarily as a longitudinal archive of prior physiological status rather than a real-time marker of immediate therapeutic response. Accordingly, hair mineral analysis may be most useful as a retrospective biomarker of chronic stress exposure. Future longitudinal studies with larger cohorts and longer follow-up are needed to determine the timeframe over which these mineral signatures change after successful anxiolytic intervention. 

## Data Availability

The datasets generated during the current study are available from the corresponding author on reasonable request.
